# Stool is a sensitive and noninvasive source of DNA for monitoring expansion in repeat expansion disease mouse models

**DOI:** 10.1242/dmm.049453

**Published:** 2022-05-13

**Authors:** Xiaonan Zhao, Cassandra McHugh, Sydney R. Coffey, Diego Antonio Jimenez, Elizabeth Adams, Jeffrey B. Carroll, Karen Usdin

**Affiliations:** 1 Section on Gene Structure and Disease, Laboratory of Cell and Molecular Biology, National Institute of Diabetes, Digestive and Kidney Diseases, National Institutes of Health, Bethesda, MD 20892, USA; 2 Behavioral Neuroscience Program, Psychology Department, Western Washington University, Bellingham, WA 98225, USA

**Keywords:** Fragile X-related disorder, Huntington's disease, Spinocerebellar ataxia types 1 and 2, Somatic instability, Stool, Peripheral DNA

## Abstract

Repeat expansion diseases are a large group of human genetic disorders caused by expansion of a specific short tandem repeat tract. Expansion in somatic cells affects age of onset and disease severity in some of these disorders. However, alleles in DNA derived from blood, a commonly used source of DNA, usually show much less expansion than disease-relevant cells in the central nervous system in both humans and mouse models. Here we examined the extent of expansion in different DNA sources from mouse models of the fragile X-related disorders, Huntington's disease, spinocerebellar ataxia type 1 and spinocerebellar ataxia type 2. We found that DNA isolated from stool is a much better indicator of somatic expansion than DNA from blood. As stool is a sensitive and noninvasive source of DNA, it can be useful for studies of factors affecting the risk of expansion, or the monitoring of treatments aimed at reducing expansion in preclinical trials, as it would allow expansions to be examined longitudinally in the same animal and allow significant changes in expansion to be observed much earlier than is possible with other DNA sources.

## Abstract (precis)

**Summary:** Stool is a readily available, noninvasive and sensitive source of DNA for monitoring repeat expansion in mouse models of four different repeat expansion diseases.

## INTRODUCTION

More than 40 genetic disorders are caused by expansion of a single short tandem repeat (STR) tract. These disorders are known as repeat expansion diseases (REDs), and include the fragile X-related disorders (FXDs) caused by expanded CGG/GCC repeats, and a large number of degenerative disorders caused by expanded CAG/CTG repeats such as Huntington's disease (HD), myotonic dystrophy type 1 (DM1) and many spinocerebellar ataxias (SCAs). The length of the repeat tract that is inherited is a major determinant of the age of onset of many REDs. However, these STRs tend to increase in repeat number both on intergenerational transmission and somatically. The extent of expansion increases with time but varies with cell type, with some cell types acquiring extremely large expansions in a relatively short period of time, and others remaining relatively stable for many years. Interest in somatic instability in the CAG/CTG repeat expansion diseases has been driven in part by studies showing that the tendency to expand somatically is associated with an earlier age at disease onset and/or more severe symptoms in HD (Swami et al., 2009; GeM_HD Consortium, 2019) and DM1 (Morales et al., 2020). In addition, genome-wide association studies (GWAS) have demonstrated that variants in mismatch repair (MMR) proteins and the structure-dependent endonuclease Fanconi-associated nuclease 1 (FAN1) are associated with pronounced variation in the age of disease onset in HD mutation carriers (GeM_HD Consortium, 2015; Hong et al., 2021). Similar results have been observed in several forms of SCA caused by CAG/CTG repeat expansions (Bettencourt et al., 2016). Similarly, loss of MSH2, an important MMR protein, has been shown to modify the timing of early disease onset in a mouse model of HD (Wheeler et al., 2003). Since mutations in MMR genes are associated with variations in the extent of somatic expansion in both patients (GeM_HD Consortium, 2019; Bettencourt et al., 2016; Flower et al., 2019; Kim et al., 2020) and mouse models (reviewed in Zhao et al., 2021a; Wheeler and Dion, 2021), this adds weight to the idea that somatic expansion is an important modifier of disease risk and/or severity. This has led to interest in pharmacological strategies that are focused on reducing somatic instability, a strategy that may be therapeutically useful for many of these diseases (Wiggins and Feigin, 2021).

Blood is generally used for measuring the repeat size in patients. However, blood cells show less somatic instability than disease-relevant cells, such as striatal neurons from both patients and mouse models of different repeat expansion diseases (Telenius et al., 1994; Takano et al., 1996; Pinto et al., 2020; Zhao et al., 2019; Kacher et al., 2021). A source of peripheral DNA that shows more extensive expansions would expedite testing of genetic factors affecting expansion risk and would be useful for monitoring therapeutic efforts to reduce expansions, at least in preclinical models. Previously we showed that expansion in the DNA derived from the small intestine is more extensive than in DNA from other tissues in a FXD mouse model (Zhao et al., 2018), and is sensitive to mutations in genetic factors like FAN1 that are important modifiers of expansion risk in patient cohorts and in mice (GeM_HD Consortium, 2015, 2019; Kim et al., 2020; Bettencourt et al., 2016; Ciosi et al., 2019; Mergener et al., 2020; Zhao et al., 2018, 2021b). Since the murine intestinal epithelium turns over every 3-4 days (Hughes et al., 1958; Walker and Leblond, 1958), large numbers of exfoliated cells from the intestine accumulate in stool. This makes mouse stool a useful source of genomic DNA for genotyping (Broome et al., 1999; Kalippke et al., 2009). Most of the exfoliated cells found in stool are epithelial cells from the colon (Iyengar et al., 1991). If the colonic epithelium is as prone to expansion as the cells of the small intestine, large expansions should also be detectable in stool samples. Here we show that the colon does show extensive expansions in mouse models of four different repeat expansion diseases, and these expansions are mirrored in the DNA isolated from stool.

## RESULTS

### Expansion in colon and stool DNA is higher than DNA from most other sources in an FXD mouse model

As with many other comparable studies, we used mice with larger repeat numbers than those present in the inherited allele in some of the repeat expansion diseases. However, the available evidence suggests that the same genetic factors affect the expansion of both longer and shorter repeats (reviewed in Zhao et al., 2019), and by using animals with larger repeat numbers, significant expansions can be observed within an experimentally reasonable time frame.

**Fig. 1.**
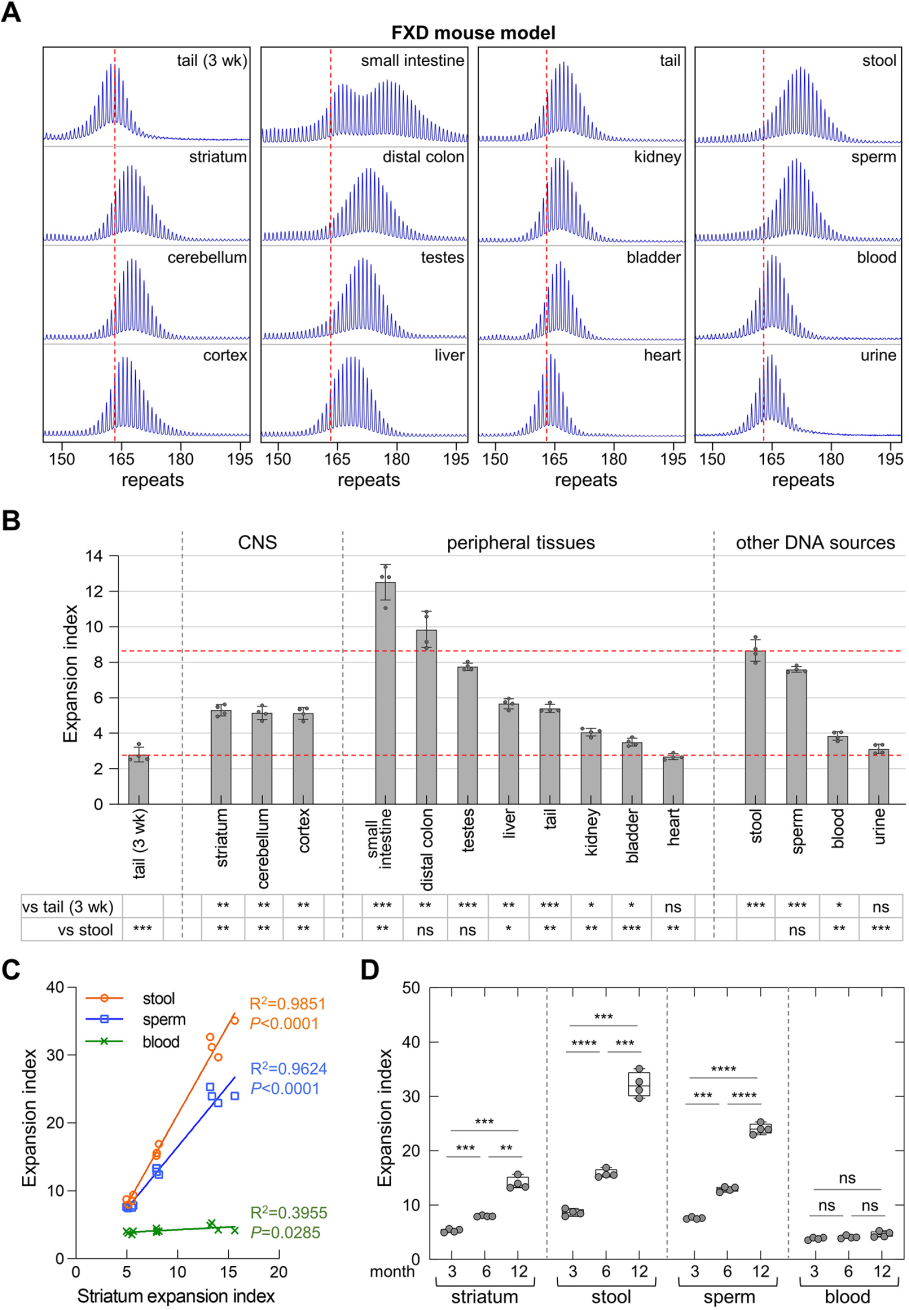
**Quantitative analyses of CGG repeat expansions in FXD mice.** (A) Typical repeat PCR profiles from tail DNA taken at 3 weeks (3 wk) and different sources of DNA collected at 3 months from the same FXD mouse with 163 repeats. The dashed lines represent the sizes of the original inherited alleles as ascertained from the tail DNA taken at 3 weeks. (B) Comparison of the expansion index (EI) in different organs and DNA sources of 3-month-old FXD mice with an average of 163 repeats in the original allele. The lower dashed line represents the basal expansion level as ascertained from the tail DNA taken at 3 weeks. The upper dashed line represents the expansion level in stool. The data represent the average of four male mice with 160-164 repeats. The error bars indicate the standard deviations of the mean. Each dot represents one animal. The EIs in different DNA sources were compared to the EI in stool and tail DNA taken at 3 weeks using a repeat measures (RM) one-way ANOVA with correction for multiple testing as described in the Materials and Methods. The adjusted *P*-values are listed in the table below. *P<0.05; **P<0.01; ***P<0.001; ns, not significant. (C) Correlation between EI in striatum and EI in stool, sperm and blood of 12 male FXD mice with 158-164 repeats at different ages. (D) Box plot of the EI of male FXD mice at different ages. The box represents the 25-75th percentiles, and the median is indicated. The whiskers show the minimum and maximum values. Each age group includes four animals with 158-164 repeats. Each dot represents one animal. The significance of the age effect was assessed using RM two-way ANOVA with correction for multiple testing as described in the Materials and Methods. **P<0.01; ***P<0.001; ****P<0.0001; ns, not significant.

We collected DNA samples from FXD mice with ∼163 inherited repeats at 3 months of age, including from the colon and stool, as well as from urine and sperm. We then compared the extent of repeat expansion using the expansion index (EI) metric (Miller et al., 2020). The EI of tail samples taken at 3 weeks of age was used as the baseline. As shown in Fig. 1, DNA from the central nervous system (CNS), liver and tail showed a ∼2-fold increase in the EI at 3 months of age. The EI in testes and sperm was somewhat higher. The similarity in the EI in these two DNA sources is consistent with our previous demonstration that most expansion in the testis is confined to the gametes (Zhao and Usdin, 2018b). The small intestine, distal colon and stool samples showed the highest levels of expansion, with the colon and stool showing very similar expansion profiles. This similarity is consistent with the fact that epithelial cells are the most common cell in the postnatal colon, and are the cells most likely to be sloughed off into the intestinal lumen and thus present in stool. In contrast, as can be seen in Fig. 1B, blood and urine samples showed little or no significant change in the EI relative to tail DNA taken at 3 weeks. Most DNA isolated from urine has been shown to be derived from the epithelial cells from the kidneys and bladder (Abedini et al., 2021). Thus, not all epithelial cells were equally expansion prone. Since the yield of DNA from mouse urine was generally poor, we did not test this source of DNA further.

The correlation with the EI in striatum was much stronger for stool and sperm than for blood (Fig. 1C). Furthermore, as can be seen in Fig. 1D, although expansions in the striatum, stool and blood increased significantly with time, the amount of expansion in blood showed relatively little change between 3 and 12 months. Thus, sperm and stool samples were very sensitive indicators of expansion. However, stool has the advantage that not only was it found to be a slightly more sensitive indicator of expansion than sperm, but it can also be used to examine expansion in both males and females.

### Expansion detected in stool DNA can be seen at an early age and increases with age and inherited repeat size in an FXD mouse model

**Fig. 2.**
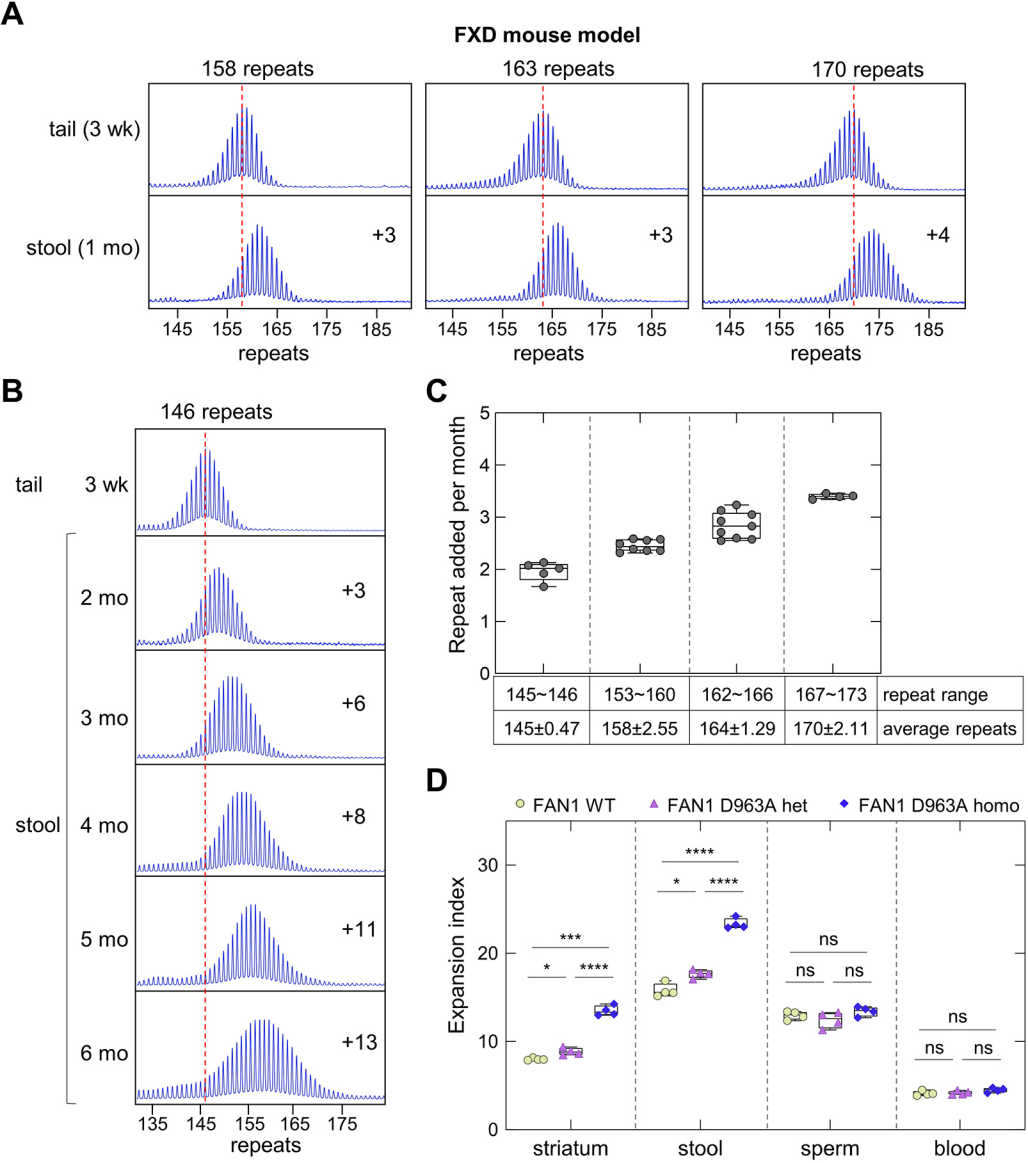
**Quantitative analyses of CGG repeat expansions in stool from FXD mice of different inherited allele sizes and genetic conditions.** (A) Typical repeat PCR profiles from tail DNA taken from a 3-week-old (3 wk) mouse, and stool samples collected from the same FXD mouse at 1 month (1 mo). The numbers associated with some of the profiles indicate the number of repeats added during the lifetime of the mouse. The dashed lines represent the sizes of the original inherited alleles as ascertained from the tail DNA taken at 3 weeks. (B) Typical repeat PCR profiles from tail DNA taken at 3 weeks, and stool samples collected from the same FXD mouse over time. The numbers associated with the profiles indicate the number of repeats added during the lifetime of the mouse. The dashed lines represent the sizes of the original inherited alleles as ascertained from the tail DNA taken at 3 weeks. (C) Repeats added per month in male FXD mice with different repeat numbers, including five mice with 145-146 repeats, eight mice with 153-160 repeats, nine mice with 162-166 repeats, and four mice with 167-173 repeats. Each dot represents one animal. The repeat size range and average repeat size are listed in the table below. (D) Box plot of the EI in the striatum and other sources DNA from 6-month-old FAN1 WT and FAN1 D963A mutant mice with an average of 161 repeats in the original allele. The data for each genotype are based on four animals with 159-164 repeats. Each dot represents one animal. In box and whisker plots, the box represents the 25-75th percentiles, and the median is indicated. The whiskers show the minimum and maximum values. The significance of the genotype effects was assessed using RM two-way ANOVA with correction for multiple testing as described in the Materials and Methods. *P<0.05; ***P<0.001; ****P<0.0001; ns, not significant.

To examine the extent of repeat expansion in the DNA from mouse stool, we collected fresh stool samples from FXD mice at different ages. Expansions were evident even in 1-month-old mice with 158+ repeats, with the repeat tract in stool DNA already being three to four repeats larger than it is in tail DNA, a DNA source that showed only modest expansions (Fig. 2A). Furthermore, even at this age, a modest effect of inherited allele size on the rate of repeat addition could be seen in mice with 170 repeats showing a gain of four repeats, compared to a gain of three repeats for mice with 158 and 163 repeats. As can be seen in Fig. 2B, even mice with only 146 repeats gained three repeats in DNA from stool by 2 months of age, and alleles detected in stool DNA continued to gain repeats over a 6-month period at a relatively consistent rate, resulting in the gain of 13 repeats relative to the tail DNA isolated from tail samples taken at 3 weeks of age. This corresponded to an average increase of two repeats per month, which increased to ∼3.5 repeats per month in mice with an inherited allele with 170 repeats (Fig. 2C).

### Expansion in stool DNA reflects the effect of FAN1 on somatic expansion in an FXD mouse model

FAN1 has been identified as a genetic modifier of disease progression in a number of repeat expansion diseases (GeM_HD Consortium, 2015, 2019; Kim et al., 2020; Bettencourt et al., 2016; Ciosi et al., 2019; Mergener et al., 2020), and we previously showed that FAN1 protects against repeat expansion in an FXD mouse model (Zhao and Usdin, 2018a). Furthermore, a D963A point mutation in the nuclease domain of FAN1 nuclease was found to result in a significant increase in expansion in the striatum (Zhao et al., 2021b). Here, we show that the same mutation resulted in a significant increase in the EI in stool even in heterozygotes (Fig. 2D). In contrast, expansion in blood and sperm samples were similar in both wild-type (WT) and *Fan1* mutant mice. Thus, stool DNA reflects the effect of an important genetic modifier of expansion risk in disease-relevant cells, whereas blood DNA does not.

### Expansion in stool DNA is also a good indicator of somatic expansion in CAG/CTG repeat expansion diseases

**Fig. 3.**
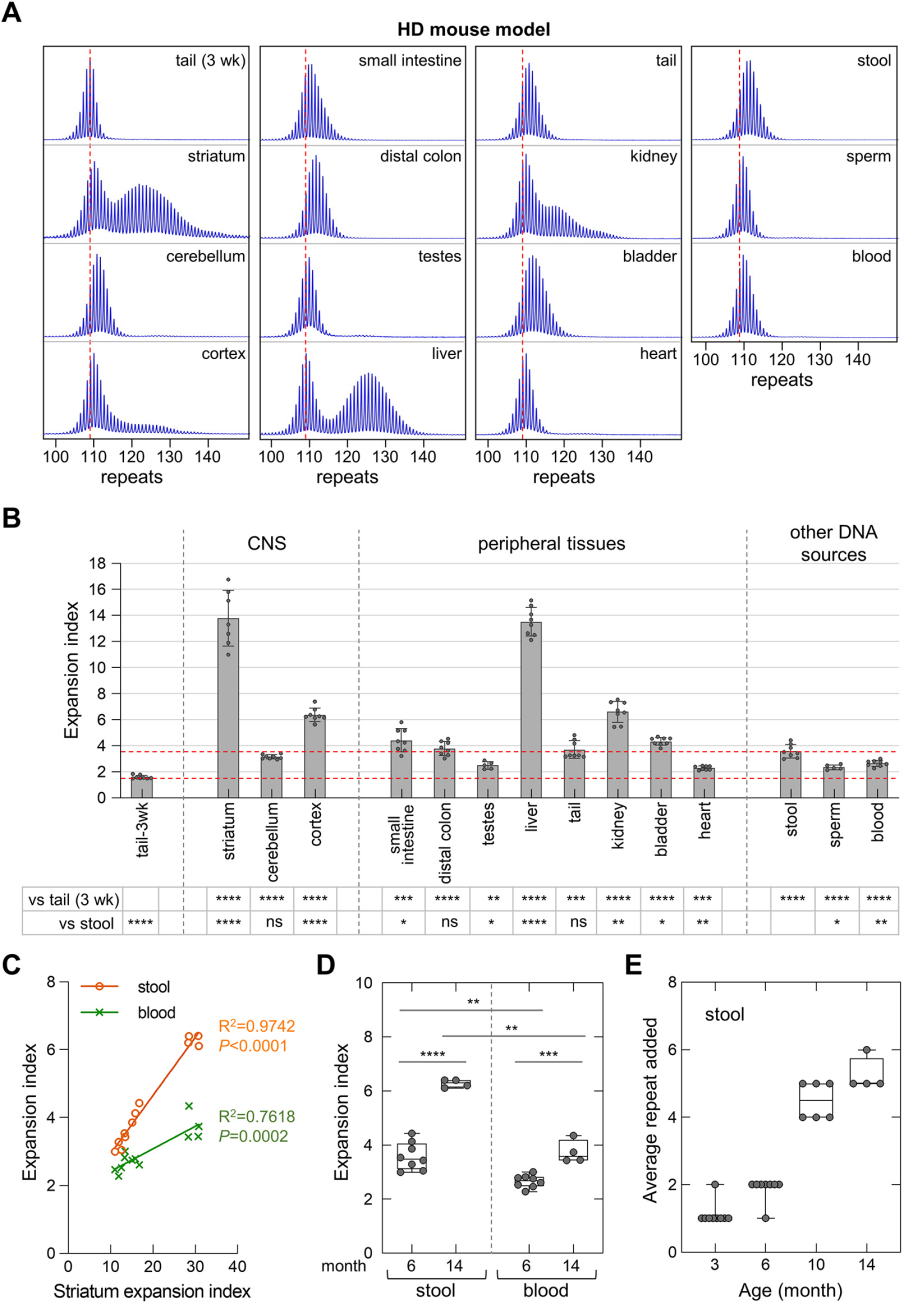
**Quantitative analyses of CAG expansions in HD mice.** (A) Typical repeat PCR profiles from tail DNA taken at 3 weeks (3 wk) and different sources of DNA collected at 6 months from the same HD mouse with 109 repeats. The dashed lines represent the sizes of the original inherited alleles as ascertained from the tail DNA taken at 3 weeks. (B) Comparison of the EI in different organs and DNA sources of 6-month-old HD mice with an average of 114 repeats in the original allele. The lower dashed line represents the basal expansion level as ascertained from the tail DNA taken at 3 weeks. The upper dashed line represents the expansion level in stool. Testes and sperm samples represent the average of five male mice with 109-124 repeats. Other data represent the average of five male and three female mice in the same repeat range. The error bars indicate the standard deviation of the mean. Each dot represents one animal. The EI in different DNA sources were compared to the EI in stool and in tail DNA taken at 3 weeks using a mixed-effects model with correction for multiple testing as described in the Materials and Methods. The adjusted *P*-values are listed in the table below. *P<0.05; **P<0.01; ***P<0.001; ****P<0.0001; ns, not significant. (C) Correlation between EI in the striatum and EI in stool or blood of 12 HD mice with 109-124 repeats at different ages. (D) Box plot of the EI in stool and blood sample in C, which were collected from five male and three female HD mice with 109-124 repeats at 6 months old, and four male HD mice with 111-113 repeats at 14 months. Each dot represents one animal. The significance was assessed using a paired (stool versus blood) or unpaired (6 months versus 14 months) two-tailed *t*-test with correction for multiple testing as described in the Materials and Methods. **P<0.01; ***P<0.001; ****P<0.0001. (E) Box plot of the repeat added in stool sample collected from HD mice with an average of 112 repeats at different ages, including nine male mice at 3 months with 110-116 repeats, five male and three female mice at 6 months with 109-124 repeats, six male mice at 10 months with 108-116 repeats, and four male mice at 14 months with 111-113 repeats. In box and whisker plots, the box represents the 25-75th percentiles, and the median is indicated. The whiskers show the minimum and maximum values. Each dot represents one animal.

To test whether stool DNA is also a good indicator of somatic expansion in other repeat expansion disease models, we measured the extent of expansion in stool DNA from a mouse model of HD. As in the FXD mice, expansions were low in heart samples. However, as can be seen in Fig. 3, the striatum and liver were the most expansion-prone tissues in this model, followed by the kidneys and cortex. In contrast, the small intestine and distal colon had a lower EI compared with that of the FXD mouse. However, the EI in stool was similar to other tissues in the HD mouse and higher than either blood or sperm (Fig. 3B). Significant correlations were seen between the EI in the striatum and the EI in both stool and blood (Fig. 3C). However, as can be seen in Fig. 3D, the EI in stool showed a larger increase with age than that seen in blood. Thus, stool was also a better indicator of somatic expansion in HD mice than blood. On average, the expansion rate in the DNA isolated from stool of HD mice with ∼112 inherited repeats was about 0.4 repeats per month (Fig. 3E).

**Fig. 4.**
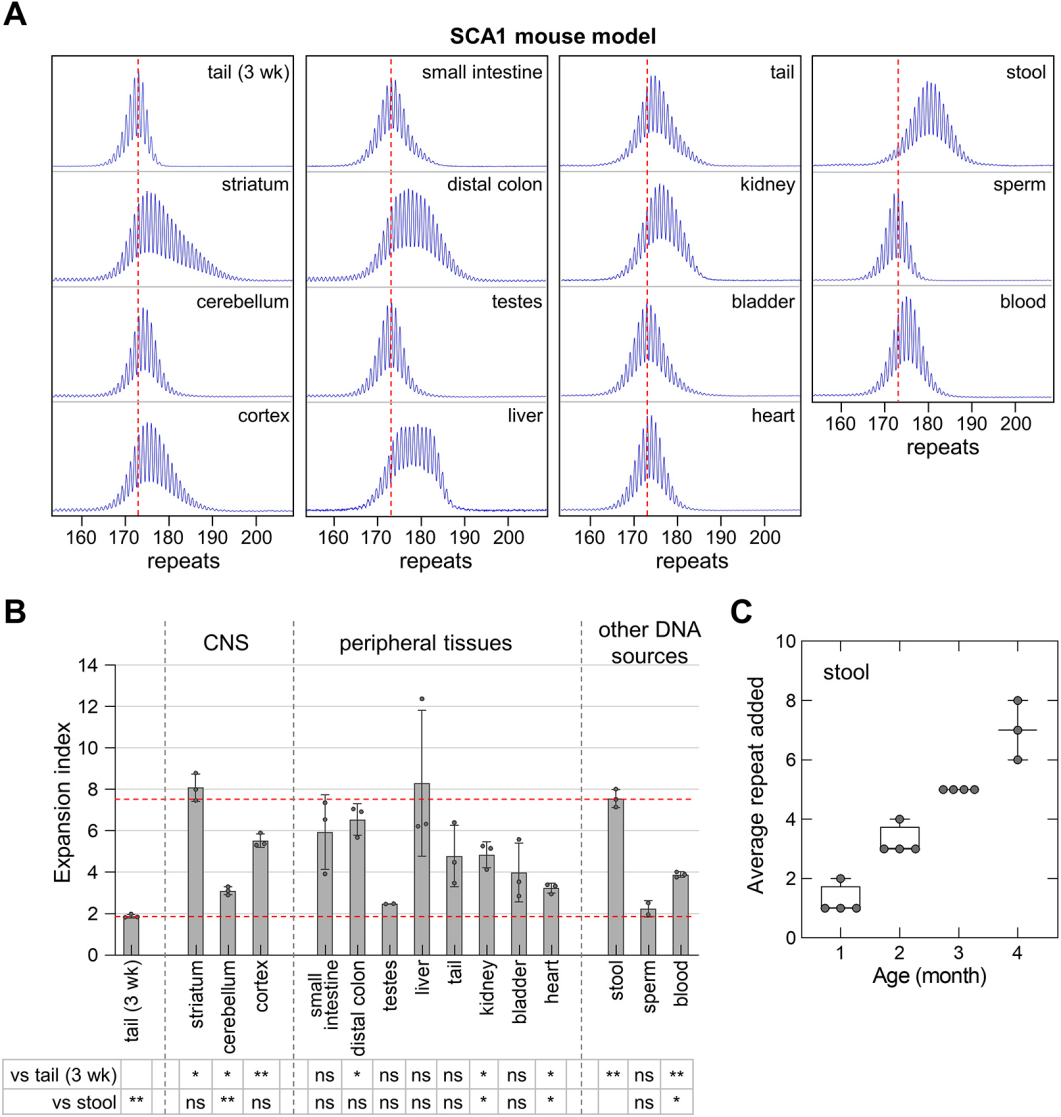
**Quantitative analyses of CAG expansions in SCA1 mice.** (A) Typical repeat PCR profiles from tail DNA taken at 3 weeks (3 wk) and different sources of DNA collected at 4 months from the same SCA1 mouse with 173 repeats. The dashed lines represent the sizes of the original inherited alleles as ascertained from the tail DNA taken at 3 weeks old. (B) Comparison of the EI in different organs and DNA sources of 4-month-old SCA1 mice with an average of 171 repeats in the original allele. The lower dashed line represents the basal expansion level as ascertained from the tail DNA taken at 3 weeks. The upper dashed line represents the expansion level in stool. Testes and sperm samples represent the average of two male mice with 171 and 173 repeats. Other data represent the average of two male and one female mice in the same repeat range. The error bars indicate the standard deviations of the mean. Each dot represents one animal. The EI in different DNA sources were compared to the EI in stool and tail DNA taken at 3 weeks using a mixed-effects model with correction for multiple testing as described in the Materials and Methods. The adjusted *P*-values are listed in the table below. *P<0.05; **P<0.01; ns, not significant. (C) Box plot of the repeat added in stool sample collected at different ages from the same animals, including two male and two female SCA1 mice with 170-176 repeats. The box represents the 25-75th percentiles, and the median is indicated. The whiskers show the minimum and maximum values. Each dot represents one animal. Only three animals were available at 4 months of age.

**Fig. 5.**
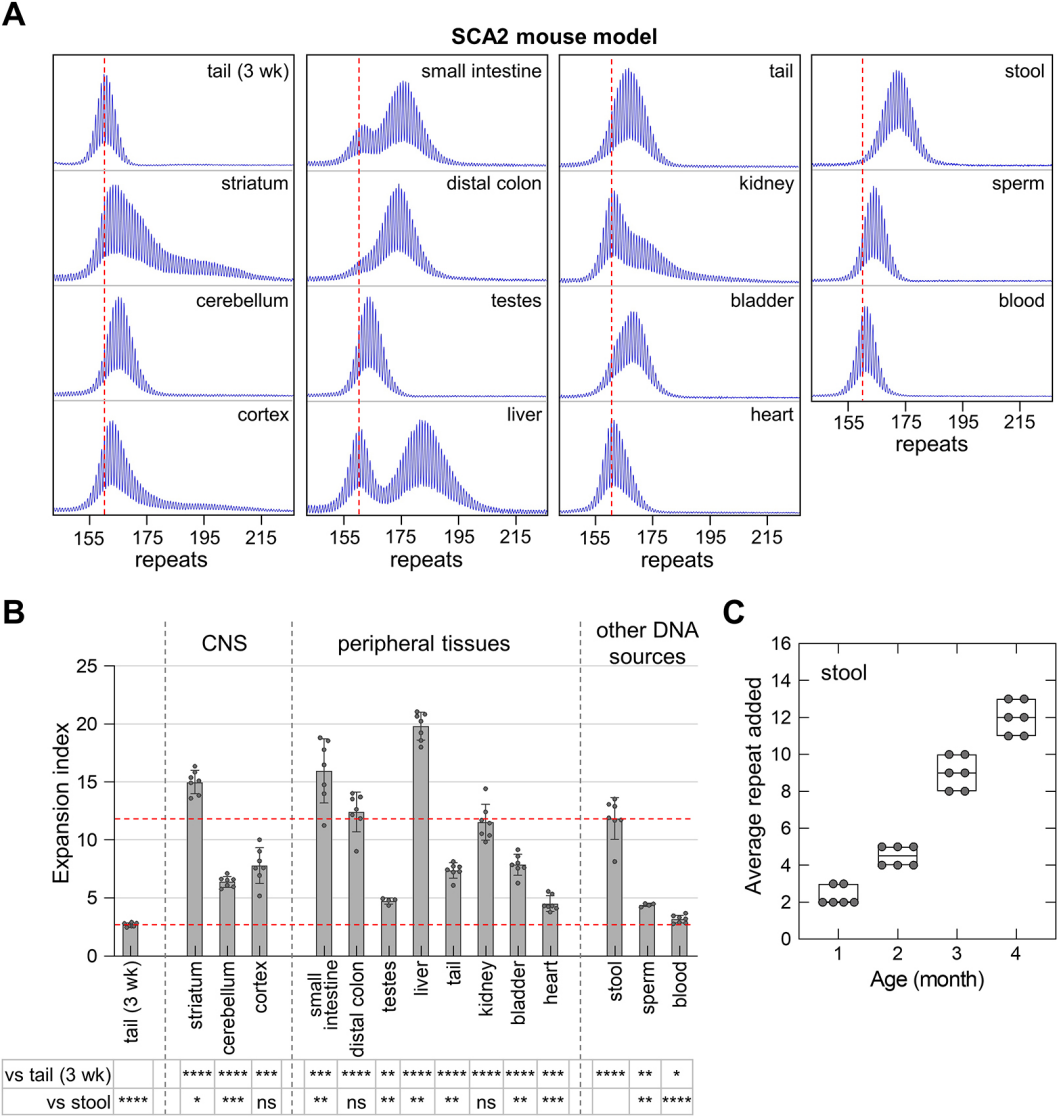
**Quantitative analyses of CAG expansions in SCA2 mice.** (A) Typical repeat PCR profiles from tail DNA taken at 3 weeks (3 wk) and different sources of DNA collected at 4 months from the same SCA2 mouse with 160 repeats. The dashed lines represent the sizes of the original inherited alleles as ascertained from the tail DNA taken at 3 weeks. (B) Comparison of the EI in different organs and DNA sources of 4-month-old SCA2 mice with an average of 158 repeats in the original allele. The lower dotted line represents the basal expansion level as ascertained from the tail DNA taken at 3 weeks. The upper dotted line represents the expansion level in stool. Testes and sperm samples represent the average of four male mice with 154-162 repeats. Other data represent the average of four male and two female mice in the same repeat range. The error bars indicate the standard deviations of the mean. Each dot represents one animal. The EI in different DNA sources were compared to the EI in stool and tail DNA taken at 3 weeks using a mixed-effects model with correction for multiple testing as described in the Materials and Methods. The adjusted *P*-values are listed in the table below. *P<0.05; **P<0.01; ***P<0.001; ****P<0.0001; ns, not significant. (C) Box plot of the repeat added in stool sample collected at different age from the same animals including four male and two female SCA2 mice with 154-162 repeats at different age. The box represents the 25-75th percentiles, and the median is indicated. The whiskers show the minimum and maximum values. Each dot represents one animal.

We also examined expansion in two other CAG/CTG repeat expansion diseases, SCA1 and SCA2. As can be seen in Fig. 4, for most DNA sources, the extent of expansion in SCA1 mice was similar to that seen in HD mice, with the exception of stool, for which the expansion was significantly higher. The EI in stool was similar to that in the striatum, liver, small intestine and distal colon, and higher than in other tissues including blood and sperm. Expansion in stool DNA from SCA1 mice with ∼173 inherited repeats increased with age at the rate of ∼1.5 repeats per month. In SCA2 mice, the overall extent of expansion was much higher than that seen in FXD, HD and SCA1 mice. As can be seen in Fig. 5, although the EI in stool was lower than the striatum and liver, it was still much higher than in blood and sperm. The expansion rate in stool DNA from SCA2 mice with ∼155 inherited repeats was ∼2.5 repeats per month. The high expansion in stool DNA in both SCA1 and SCA2 mouse models suggests that expansion in DNA isolated from stool is also a sensitive indicator of somatic expansion in the SCA1 and SCA2 mouse models.

## DISCUSSION

We have shown that repeat expansion can be detected in the DNA isolated from stool samples of mouse models of four different repeat expansion diseases. In the FXD (Fig. 1), SCA1 (Fig. 4) and SCA2 (Fig. 5) mouse models, the extent of expansion in mouse stool samples was higher than in blood, and comparable to or greater than expansion in the striatum. Although expansion in stool DNA in the HD mouse model was lower than that in the striatum, it was still higher than that in blood, and shows a better correlation with the extent of expansion in the striatum (Fig. 3). The extent of expansion in stool DNA is comparable to that seen in the distal colon, which is consistent with the fact that most of the host DNA isolated from stool is derived from this part of the digestive system. While colonic epithelial cells are rapidly dividing and neurons are post-mitotic, the available evidence from mouse models suggests that the genetic factors involved in generating somatic expansions are similar in both dividing and non-dividing cells (reviewed in Zhao et al., 2021a), and thus that the expansion mechanisms might also be similar. The correlation seen between the extent of expansion in stool samples and that seen in the striatum (Fig. 1C, Fig. 3C) suggests that the extent of expansion in stool DNA is a good indicator of the extent of expansion in the brain. However, since expansion is not seen in the DNA derived from urine, in which most host DNA is epithelial in origin, not all epithelial sources are equally prone to expansion.

Expansion in DNA isolated from stool is apparent at an early age, and is sensitive to inherited repeat size (Fig. 2). Importantly, expansion in stool DNA also reflects the effect of an important genetic modifier of expansion risk in the FXD mouse model (Fig. 2D). We previously showed that heterozygosity for a D963A point mutation resulted in significantly more expansions in the striatum, cerebellum and liver, but not in other tissues (Zhao et al., 2021b). As can be seen in Fig. 2D, a significant increase in the extent of expansion can also been seen in the DNA from the stool of these animals.

The propensity of any given cell type to expand likely reflects, at least in part, the balance between the levels of factors that promote expansion and those that protect against it. Notably, many organs show a similar extent of expansion in different RED mouse models. For example, heart shows little or no expansion in all the models, whereas the striatum and liver always show higher levels of expansion. This similarity would be consistent with the idea that the same genetic factors affect expansion in different mouse models. However, there is some discordance between the extent of expansion in some organs in different models. For example, the small intestine and distal colon show high levels of expansion in FXD, SCA1 and SCA2 mouse models, but not in the HD mouse model. The kidneys show more expansion in HD and SCA2 mouse models than either SCA1 or FXD mouse models, and the extent of expansion in sperm and testes is high in the FXD model but not in others. These differences might reflect differences in the level of transcription of the affected gene or some other effect of the flanking sequences.

Notably in all four mouse models, expansion in the CNS is highest in the striatum, even though the pathology of the SCAs primarily involves the cerebellum. However, the relatively low level of expansion in the cerebellum mirrors what is seen in SCA1 and SCA2 patients (Pinto et al., 2020; Chong et al., 1995; Lopes-Cendes et al., 1996; Zühlke et al., 1997; Hashida et al., 1997; Matsuura et al., 1999). Thus, the cells that accumulate the largest expansions are not necessarily those that are the most vulnerable to the downstream consequences of expansion. This parallels the observation that tissues expressing the highest levels of the pathogenic protein responsible for these diseases are not always the sites of greatest pathology either (Sharp et al., 1995). It might be that some cells are particularly sensitive to the toxic effects of the mutant protein. In those cells, the addition of a small number of repeats could have a significant effect.

HD is the only disease in the group in which gastrointestinal (GI) dysfunction is a major symptom. Signs of this dysfunction can appear early, before evidence of CNS neurodegeneration is apparent (Kobal et al., 2018; Wood et al., 2008; Andrich et al., 2009). Similar symptoms are seen in mouse models of HD where they are associated with a decrease in the length of the colon (Stan et al., 2020), the mucosal thickness and villus length (van der Burg et al., 2011). While the mutant protein that is responsible for HD pathology is widely expressed in cells of the GI tract, whether dysfunction is due to GI-cell-autonomous effects is unknown. Regardless, there are many other reasons why stool might be a useful source of DNA. First, stool collection is noninvasive and quick, causing minimum stress to the animals, and each stool pellet provides enough mouse DNA for ten or more PCR assays. Second, expansions in mouse stool DNA are more extensive than in blood DNA and are more sensitive to age and repeat size. This allows expansions to be more rapidly detected in stool samples from younger animals, even those with smaller repeat numbers. It also reduces the time needed to see meaningful differences in the extent of expansion in mice with different genotypes or mice that receive different potential expansion-modifying treatments. Third, at least in the case of one important known genetic modifier of expansion risk, namely FAN1, expansion in stool DNA mirrors what is seen in the brain. Finally, the simple, rapid and noninvasive collection of stool samples allows repeat length changes to be easily and frequently monitored in the same animal over time. Thus, the use of stool DNA should expedite studies on the expansion mechanism and experimental approaches to limit these expansions. Other readily accessible sources of DNA like hair follicles might also be worth testing. However, because of low yields (Picazo and García-Olmo, 2015) and contamination risk (Cinelli et al., 2007), these may be less than ideal. Furthermore, since hair follicles from different parts of the body are comprised of cells with different embryonic origins that have different gene expression profiles (reviewed in Driskell et al., 2011), they might also show differences in the extent of expansion.

There is some evidence to suggest that the propensity of different human tissues to expand is similar to that seen in these mouse models (Pinto et al., 2020; Chong et al., 1995; Lopes-Cendes et al., 1996; Zühlke et al., 1997; Hashida et al., 1997; Matsuura et al., 1999). In addition to the higher levels of expansion in the striatum and lower levels of expansion in the cerebellum of SCA1 and SCA2 patients mentioned above, HD patients show similarly elevated levels of expansion in the striatum, as well as in tissues like the liver that show high levels of expansion in mouse models (Pinto et al., 2020; Kennedy et al., 2003; Swami et al., 2009; Telenius et al., 1994; De Rooij et al., 1995). This raises the possibility that human stool samples could also show more extensive expansions than in blood. Although more work is required to properly evaluate the clinical use of somatic expansion as a measure of disease progression/onset risk, our results might have important clinical implications as stool might be more useful than blood for the assessment of somatic expansion risk. Stool might also be useful for monitoring the efficacy of clinical trials of therapies aimed at reducing somatic expansion. Furthermore, the similarity we have seen in the cell-type specificity of expansion in the four disease models suggests that stool could be a useful peripheral source of DNA for monitoring expansions in patients with other repeat expansion diseases and/or their mouse models.

## MATERIALS AND METHODS

### Reagents

All reagents were from Sigma-Aldrich (St Louis, MO, USA) unless otherwise specified. Primers were from Life Technologies (Grand Island, NY, USA). Capillary electrophoresis of fluorescently labeled PCR products was carried out by the Roy J Carver Biotechnology Center, University of Illinois (Urbana, IL, USA).

### Mouse generation, breeding and maintenance

The generation of FXD and *Fan1* D963A mice was described previously (Entezam et al., 2007; Zhao et al., 2021b). These mice were maintained at the National Institutes of Health (NIH) in a manner consistent with the Guide for the Care and Use of Laboratory Animals (NIH publication no. 85-23, revised 1996) and in accordance with the guidelines of the National Institute of Diabetes and Digestive and Kidney Diseases (NIDDK) Animal Care and Use Committee, who approved this research (ASP-K021-LMCB). The generation of the HD (Wheeler et al., 1999), SCA1 (Watase et al., 2002) and SCA2 (Sen et al., 2019) mouse models was described previously. HD mice, SCA1 mice (provided by Huda Zoghbi, Howard Hughes Medical Institute, Baylor College of Medicine, Houston, TX, USA) and SCA2 mice (provided by Georg Auburger, Goethe University Medical School, Frankfurt, Germany) were maintained at Western Washington University (WWU) in a manner consistent with protocols approved by the WWU institutional animal care and use committee. All mice are on a C57BL/6J background.

### Mouse stool and urine sample collection

For stool collection, mice were moved to a clean cage with a mat. Three to five pieces of fresh stool were collected in a 1.5 ml tube and transferred to dry ice. Stool samples were kept at −80°C until further processing. Mouse urine was collected in one of two ways. The first involved holding the animal over a 1.5 ml collection tube while lightly stroking its belly, and the second involved placing the mouse in a clean, dry, empty cage covered with a plastic wrap until it urinated. The urine was then aspirated with a pipette and transfered to the collection tube. These steps were repeated until at least 300 μl of urine was collected. Urine samples were kept at 4°C for up to 24 h before processing, or at −80°C until further processing.

### DNA isolation

DNA from the tails of 3-week-old mice was extracted for genotyping using the KAPA Mouse Genotyping Kit (KAPA Biosystems, Wilmington, MA, USA). A 5 cm region of the jejunum starting 10 cm downstream of the stomach was used as the small intestine sample. A 5 cm region of the colon upstream of the anus was used as the distal colon sample. DNA from tissue samples was isolated using a Maxwell^®^16 Mouse Tail DNA Purification Kit (Promega, Madison, WI, USA) according to the manufacturer's instructions. DNA from blood was isolated using a Maxwell^®^16 Blood DNA Purification Kit (Promega, Madison, WI, USA) according to the manufacturer's instructions. Sperm collection and DNA preparation were as previously described (Zhao and Usdin, 2018b). DNA from stool was isolated using a Norgen Stool DNA Isolation Kit (Norgen Biotek, Thorold, Ontario, Canada) according to the manufacturer's instructions. A single mouse stool pellet weighed an average of 30.8 mg and yielded ∼5.5 µg of DNA. This was sufficient for >10 repeat PCR assays (see below). DNA from urine was isolated using a Norgen Urine DNA Isolation Micro Kit (Norgen Biotek, Thorold, Ontario, Canada) according to the manufacturer's instructions.

### Genotyping and analysis of repeat number

Repeat size analysis of the *Fmr1*, *Atxn1*, *Atxn2* and *Htt* alleles in the FXD, SCA1, SCA2 and HD mice, respectively, was carried out using a fluorescent PCR assay with fluorescein amidite (FAM)-labeled primer pairs. The primers FAM-labeled FraxM4 (FAM-5′-CTTGAGGCCCAGCCGCCGTCGGCC-3′) and FraxM5 (5′-CGGGGGGCGTGCGGTAACGGCCCAA-3′) were used for the *Fmr1* allele (Entezam et al., 2007), the primers FAM-labeled 8930 (FAM-5′-CAGACGCCGGGACACAAG-3′) and 8931 (5′-ATCATCGTCTGATGGGGATG-3′) were used for the *Atxn1* allele (Watase et al., 2002), the primers FAM-labeled SCA2Ex1-Fwd5 (FAM-5′-CCCCGCCCGGCGTGCGAGCCGGTGTAT-3′) and SCA2Ex1-Rev2 (5′-CGGGCTTGCGGCCAGTGG-3′) were used for the *Atxn2* allele (Sen et al., 2019), and the primers FAM-labeled CAG1 (FAM-5′-ATGAAGGCCTTCGAGTCCCTCAAGTCCTTC-3′) and HU3 (5′-GGCGGCTGAGGAAGCTGAGGA-3′) were used for the *Htt* allele (Lee et al., 2010). The amount of DNA template in the PCR mix varied for different samples. For blood, sperm and tissue samples, 100 ng of DNA was used as a template. For stool samples, 200-400 ng of DNA was used as a template, as DNA isolated from stool contains a large amount of microbial DNA. For urine samples, 10-50 ng of DNA was used as a template, as the DNA yield varied between urine samples from different animals. The PCR mix for the *Fmr1*, *Atxn1* and *Atxn2* alleles contained 2 μl DNA template, 1× KAPA2G Fast HotStart Genotyping Mix (KAPA Biosystems, Wilmington, MA, USA), 2.4 M betaine, 2% DMSO and 0.5 μM each of the primers. PCR parameters for the *Fmr1* allele were: 95°C for 10 min; 35 cycles of 95°C for 30 s, 65°C for 30 s and 72°C for 90 s; followed by incubation at 72°C for 10 min. PCR parameters for the *Atxn1* allele were: 95°C for 10 min; 40 cycles of 95°C for 30 s, 58°C for 30 s and 72°C for 90 s; followed by incubation at 72°C for 10 min. PCR parameters for the *Atxn2* allele were: 95°C for 10 min; 40 cycles of 95°C for 40 s, 60°C for 40 s and 72°C for 90 s; followed by incubation at 72°C for 10 min. The PCR mix for the *Htt* allele contained 2 μl DNA template, 1× KAPA2G Fast HotStart Genotyping Mix (KAPA Biosystems, Wilmington, MA, USA), 1.2 M betaine, 1% DMSO and 0.5 μM each of the primers. Touchdown PCR was used to amplify the *Htt* allele with the following parameters: 95°C for 10 min; 10 cycles of 95°C for 30 s, 72°C with −1°C/cycle for 30 s and 72°C for 90 s; 28 cycles of 95°C for 30 s, 63°C for 30 s and 72°C for 90 s; followed by incubation at 72°C for 10 min. The PCR products were resolved by capillary electrophoresis on an ABI Genetic Analyzer. The resultant fsa file was then displayed using a previously described custom R script (Hayward et al., 2016) that is available upon request.

The number of repeats in the modal allele found in tail samples collected from 3-week-old mice was used as an indicator of the number of original inherited repeats for all samples collected from that animal. For tissues showing a unimodal distribution of allele sizes, the difference between the repeat number present in the modal allele and the repeat number of the modal allele in tail DNA from 3-week-old mice was used as a measure of the extent of expansion. For tissues with a bimodal distribution of alleles, the extent of expansion was calculated by subtracting the repeat number in tail DNA from 3-week-old mice from the number of repeats of the modal allele in the larger of the two allele populations. The expansion rate was calculated based on data from an average of at least two different time points between 2 to 12 months from the same animal. We also quantified somatic expansions using the EI as a metric (Miller et al., 2020). The EI of tail samples taken from 3-week-old mice was used as the baseline.

### Statistical analyses

Statistical analyses were performed using GraphPad Prism 9.3. For comparisons of EI in different samples to EI in stool or tail DNA taken at 3 weeks, statistical significance was assessed using either a mixed-effects model when not all organs were available for all animals, or a repeated measures (RM) one-way ANOVA, both with Geisser–Greenhouse correction and Dunnett's correction for multiple comparisons. For comparisons of EI in samples with different ages or different genotypes, statistical significance was assessed using the RM two-way ANOVA with Geisser-Greenhouse correction and Tukey's correction for multiple comparisons. For comparisons of EI in stool and blood at different ages, statistical significance was assessed using paired (stool versus blood) or unpaired (6 months versus 14 months) two-tailed *t*-test with Holm–Sidak's correction for multiple comparisons. Correlation between EI of different tissues was assessed using linear regression.
